# Lessons from dynamic cadaver and invasive bone pin studies: do we know how the foot really moves during gait?

**DOI:** 10.1186/1757-1146-2-18

**Published:** 2009-05-27

**Authors:** Christopher J Nester

**Affiliations:** 1 Centre for Health, Sport and Rehabilitation Research, University of Salford, UK

## Abstract

**Background:**

This paper provides a summary of a Keynote lecture delivered at the 2009 Australasian Podiatry Conference. The aim of the paper is to review recent research that has adopted dynamic cadaver and invasive kinematics research approaches to better understand foot and ankle kinematics during gait. It is not intended to systematically cover all literature related to foot and ankle kinematics (such as research using surface mounted markers). Since the paper is based on a keynote presentation its focuses on the authors own experiences and work in the main, drawing on the work of others where appropriate

**Methods:**

Two approaches to the problem of accessing and measuring the kinematics of individual anatomical structures in the foot have been taken, (i) static and dynamic cadaver models, and (ii) invasive in-vivo research. Cadaver models offer the advantage that there is complete access to all the tissues of the foot, but the cadaver must be manipulated and loaded in a manner which replicates how the foot would have performed when in-vivo. The key value of invasive in-vivo foot kinematics research is the validity of the description of foot kinematics, but the key difficulty is how generalisable this data is to the wider population.

**Results:**

Through these techniques a great deal has been learnt. We better understand the valuable contribution mid and forefoot joints make to foot biomechanics, and how the ankle and subtalar joints can have almost comparable roles. Variation between people in foot kinematics is high and normal. This includes variation in how specific joints move and how combinations of joints move. The foot continues to demonstrate its flexibility in enabling us to get from A to B via a large number of different kinematic solutions.

**Conclusion:**

Rather than continue to apply a poorly founded model of foot type whose basis is to make all feet meet criteria for the mechanical 'ideal' or 'normal' foot, we should embrace variation between feet and identify it as an opportunity to develop patient-specific clinical models of foot function.

## Background

Thankfully, in biomechanics terms, we no longer view the foot as the triangle at the bottom of the leg. The term "foot" suggests that some single functional entity exists, when in fact the 26 bones, hundreds of ligaments and muscles demands that we adopt a far more complex conceptual and experimental model of the foot. In any discussion of foot biomechanics, the foot is traditionally broken into at least two parts, the rearfoot and midfoot, tending to focus on the subtalar and midtarsal joints. In the last decade multi-segment foot models have provided new insight into how the small and often assumed minor articulations in the foot move [1-5]. This can help inform both our understanding of 'normal' foot biomechanics, upon which much of clinical and surgical practice is based, and the development of clinical and experimental hypotheses as to how pathology occurs. This links directly to how foot orthoses, footwear and surgery might be best used to elicit a biomechanical effect and subsequent clinical response.

However, multi-segment foot models have their own limitations. Inevitably, the skin to which markers are attached moves relative to the underlying bones they are intended to represent. More critically, we cannot reliably measure the motion of each individual bone in the foot. This may result in incorrect extrapolation from multi segment foot model data about the kinematics of joints that comprise a segment within that model.

The perfect experimental (and clinical) scenario is that we are able to directly measure the kinematics of the individual bones of the foot. Access to tissues of the foot would provide real insight and quickly enable to us test many clinical hypotheses that have persisted despite a lack of evidence to substantiate them. Advancement in our understanding could be greatly accelerated. In coming years, dynamic imaging techniques may offer excellent solutions to the challenge of measuring the performance of individual structures in the foot (though these will have their own limitations) but until these are available we must rely on direct physical contact with structures of the foot as the best means of measuring foot motion.

## Methods

Two approaches to the problem of accessing and measuring the biomechanics of individual anatomical structures in the foot have been taken, (i) static and dynamic cadaver models, and (ii) invasive in-vivo research. Cadaver models offer the advantage that there is complete access to all the tissues of the foot, not just the bones, and you can investigate other aspects of the foot that might influence its 'performance' through subsequent dissection (such as checking for the presence of arthritic changes in a joint). It is pertinent to question whether dead tissue behaves in the same manner as living tissue, but this issue seems to be regarded as an acceptable limitation if care is taken in use of the tissues. However, the greatest disadvantage is that the cadaver must be manipulated and loaded in a manner which replicates how the foot would have performed when in-vivo. Static cadaver models fail to replicate any functional task of note though they can still offer some insight into the basic function of ligaments or muscles, and soft tissue properties. Dynamic cadaver models attempt to make the cadaver feet 'walk again' but achieving this is very complex [6-16] (Additional file 1). The cadaver must be mounted on a mechanism that has as many degrees of freedom as the human body. Loads must be applied to the specimen and its tendons at a magnitude and rate as occurs in gait (or as close as possible). Moving the specimen and loading the individual tendon and tibia/foot structures must be synchronised exactly. These parameters must also be adjustable as the input data driving the dynamic model (typically tibial motion, forces applied to the tibia/plantar surface and residual tendons) is at best an average of a small number of other feet, and certainly not in-vivo data from the foot being tested.

Invasive in-vivo foot kinematics research has a long history [17-26] and it has been some of the most cited work in the field (Additional file 2). The key value of this data is its validity in describing how the bones of the foot move, but the key difficulty is how generalisable this data is to the wider population. The truth is that the data are not generalisable, but data have already proved their value by providing evidence to refute several traditional ideas of foot function. Critically, it has helped demonstrate the significant inter-subject variation that exists. This questions any notion of a model of foot function that is based on the concept of a single 'ideal' foot type, foot alignment or movement pattern. Other difficulties with an invasive approach are that in most cases access is limited to the bones, and even then, only a selection of foot bones. There is also the issue of whether participants walk normally with pins inserted and using footwear and orthoses is very difficult (though not impossible [19]). Some question the ethical basis to this research, but in fact there is a long tradition of invasive research without reports of complications in participants who follow protocol. Surgery is via minimal incision in sterile conditions and under local infiltration of anaesthesia. There are clear post study protocols for weightbearing and medical support.

## Results and discussion

### What have we learnt about foot and ankle kinematics?

**Figure 1:**
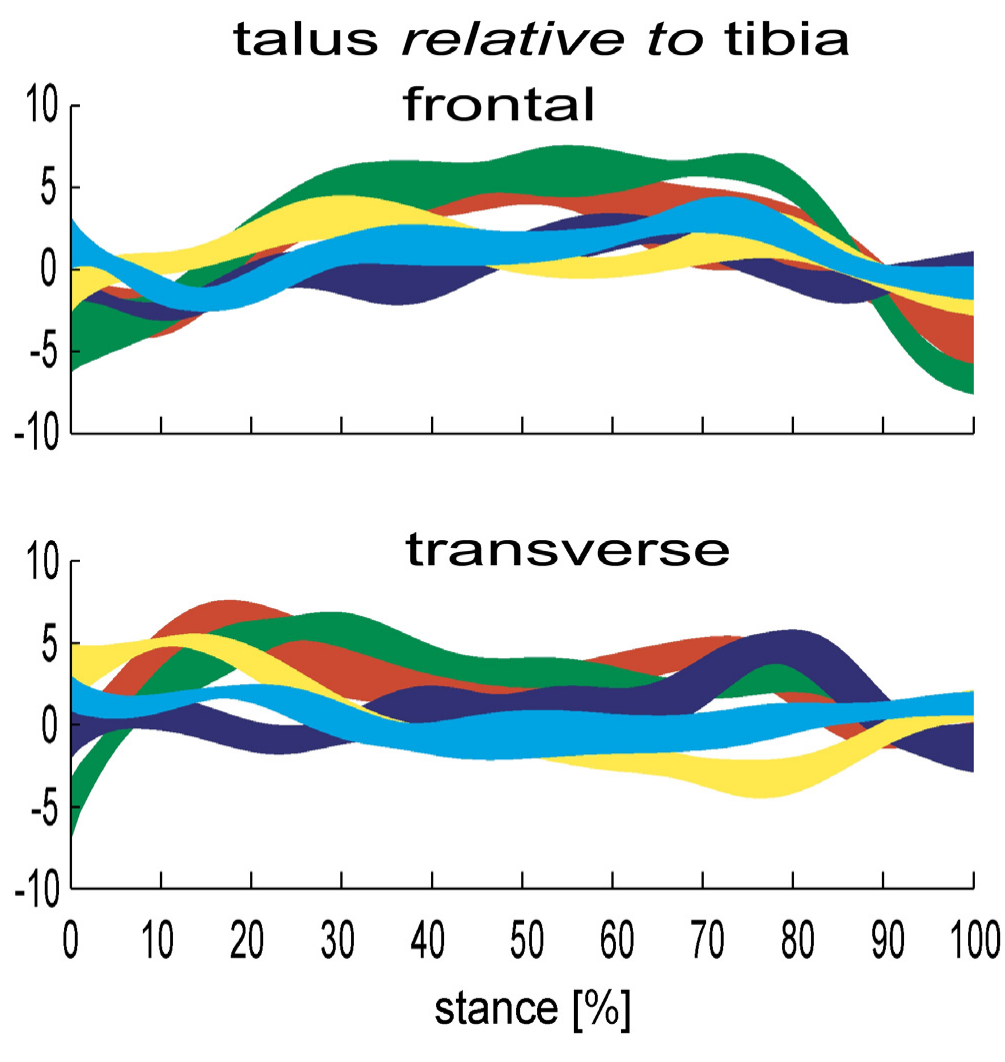
**Ankle kinematics for 5 subjects during stance (0–100%)**. Each band describes the mean +/- 1SD for each subject. +ve angles are eversion and adduction of the talus relative to tibia. Motion in degrees(°).

A key finding is the considerable freedom of movement that exists at the ankle. For the frontal and transverse planes, respectively, Lundgren et al [26] reported a mean total range of motion of 8.1° and 7.9° during walking (n = 5) (figure 1), Arndt et al [27] reported 12.2° and 8.7° in slow running (n = 4), and using a dynamic cadaver model of stance, Nester et al [9] reported a mean of 15.3°, and 10.0° (n = 13). Whilst in almost all cases the range of sagittal plane motion was greater, the ankle is certainly not limited to the role of a dorsi- and plantarflexion provider, as was traditionally thought.

Furthermore, there is clear evidence that in some feet the ankle displays more frontal and transverse plane motion than the subtalar joint, which was traditionally perceived as the rearfoot joint most able to move in these planes. In the case of transverse plane motion, Lundgren et al [26] reported that the total range of ankle motion was greater than the equivalent subtalar motion in 3 of 4 participants (in walking). Nester et al [9] reported greater transverse plane ankle motion compared to subtalar motion in 7 of 11 cadaver feet, and Arndt et al [27] reported the same in all 3 of their participants (in slow running). In the case of frontal plane motion, Arndt et al [27] found ankle motion to be greater than the equivalent frontal plane subtalar motion in 2 of 3 participants for which data was available (slow running). Lundgren reported the same in 1 of 4 participants in walking [26] as did Nester et al [9] in 8 of 11 cadavers. Based on these data, the subtalar joint is certainly not the sole 'torque converter' described in many texts, and in fact the ankle and subtalar jonts share this function, with each adopting different roles for different individuals.

The inter-subject difference in how the ankle and subtalar joints move is also evident in the pattern of movement during stance. Lundgren et al's [26] subject-specific data illustrates that some people display adduction of the talus at the ankle (5–10°) in the first 20% of stance, with other participants showing little motion at all (figure 1). Similarly in slow running [27], 2 of 4 participants showed eversion of the talus at the ankle (> 10° in first 40% of stance), the other two showing little motion at all over the same period. The variation between subjects in the frontal and transverse plane 'role' of the ankle and subtalar joints suggests they could work in tandem to provide the motion required for each person. Certainly, we should never prescribe distinctive roles to these two joints as has been the case (ankle = sagittal plane, subtalar = torque converter) and we might consider them to have quite similar functional roles in the frontal and transverse planes.

**Figure 2:**
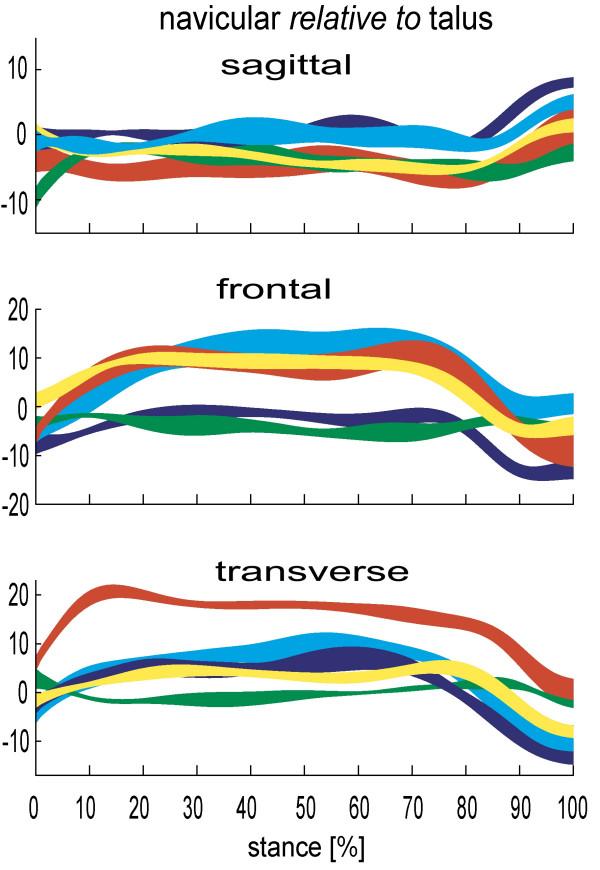
**Talo-navicular kinematics for 5 subjects during stance (0–100%)**. Each band describes the mean +/- 1SD for each subject. +ve angles are plantarflexion, eversion and adduction of the talus relative to tibia. Motion in degrees(°).

**Figure 3:**
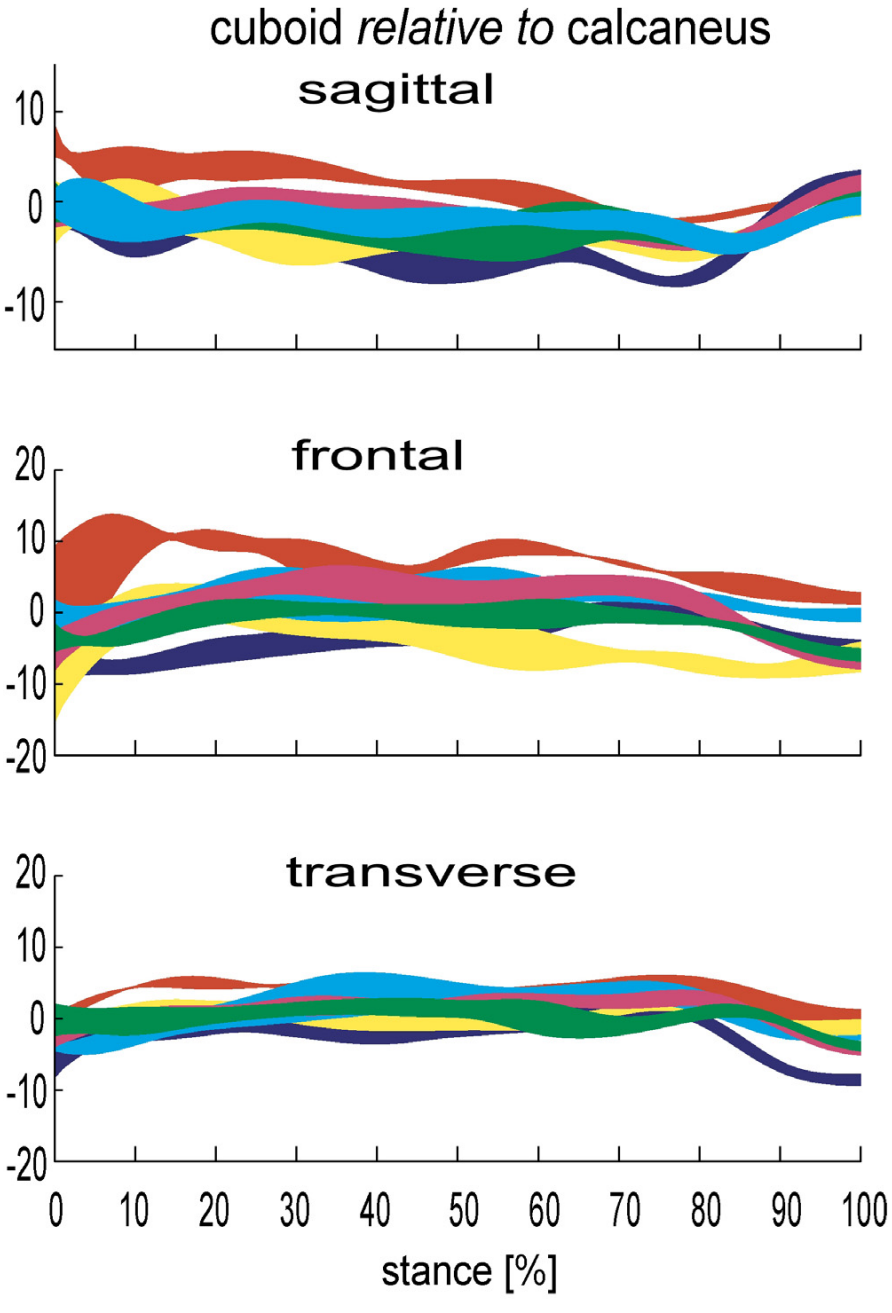
**Calcaneo-cuboid kinematics for 6 subjects during stance (0–100%)**. Each band describes the mean +/- 1SD for each subject. +ve angles are plantarflexion, eversion and adduction of the talus relative to tibia. Motion in degrees(°).

Published data consistently illustrate the significant freedom of movement at the talonavicular joint (figure 2), and to a lesser extent the calcaneocuboid joint (figure 3). In the sagittal, frontal and transverse plane respectively, Lundgren et al [26] reported 8.4° (1.1°), 14.9° (6.1°), 16.3° (6.5°) total range of motion at the talonavicular joint during walking. Arndt et al [27] reported similar sagittal and frontal plane motion in slow running, but ~50% less transverse plane motion. Given the more angular articular facets it is no surprise that the calcaneocuboid joint demonstrates less motion than the talonavicular joint in most subjects studied. However, the mean total range of calcaneocuboid motion in stance (7.8°, 6.3°, 6.9° respectively [26]) is greater than the equivalent subtalar joint motion in some in-vivo subjects [26,27] and cadaver feet [9], reinforcing its important role in overall foot function.

As with ankle and subtalar motion, there is no consistent pattern between people in the range of motion the talonavicular and calcaneocuboid joints display. For one participant of Lundgren et al [26] study, a total of 21° of motion was observed in the frontal and transverse planes during stance, yet only 5.2° and 6.0° in another participant. Remarkably, despite these stark differences, in the sagittal plane the same participants displayed 8.0° and 8.1° range of sagittal plane motion, respectively. Quite how such inter-subject variation is integrated into a clinical conceptual model of foot kinematics has yet to be determined. However, given these data are from asymptomatic feet, the data makes a mockery of any notion that a clinician should seek to alter the foot biomechanics of all patients such that their feet achieve some hypothetical mechanical ideal (i.e. one foot model fits all feet). It is far from fitting that in the year we celebrate the 150th anniversary of Darwin's 'discovery' of essential variations in nature, that foot health professionals continue to use a clinical model of foot function which seeks to eliminate all variation between our patients. Furthermore, remaining as a 'variation' of nature rather than a clone of the hypothetical 'Root' foot type is likely to be central to a person remaining symptom-free for most of their lives, since their own body will have adapted to adequately cope with its own variations.

**Figure 4:**
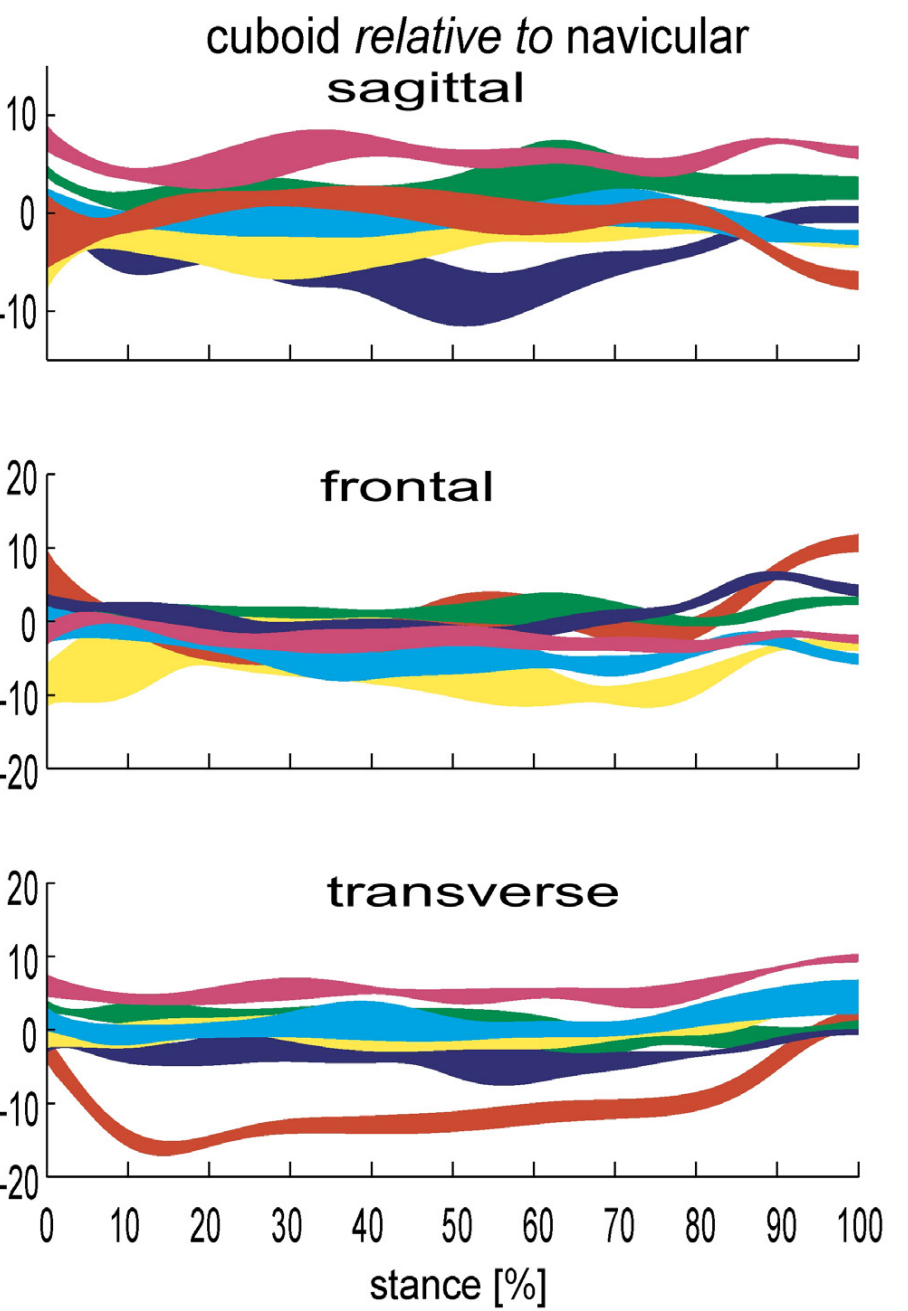
**Cuboid-navicular kinematics for 6 subjects during stance (0–100%)**. Each band describes the mean +/- 1SD for each subject. +ve angles are plantarflexion, eversion and adduction of the talus relative to tibia. Motion in degrees(°).

Many clinical models of foot biomechanics combine the navicular and cuboid, but data from Lundgren et al [26] indicates that motion between these bones is comparable or greater than that at the subtalar joint (which we never ignore) (figure 4). Identifying this capability, and the fact that motion between the medial cuneiform and navicular is equal to or greater than motion at the talonavicular joint in some feet, is perhaps one of the most important findings from the recent dynamic cadaver and invasive foot kinematic studies. This is important because data demonstrate that the tarsal bones are able to make a significant contribution to the kinematics of the overall foot. Motion that was previously attributed to the midtarsal joint and rearfoot was most likely taking place between the cuneiforms, the navicular, and cuboid. These movements are invisible clinically due to overlying tissue and consequently are completely absent from most if not all clinical models of the foot.

**Figure 5:**
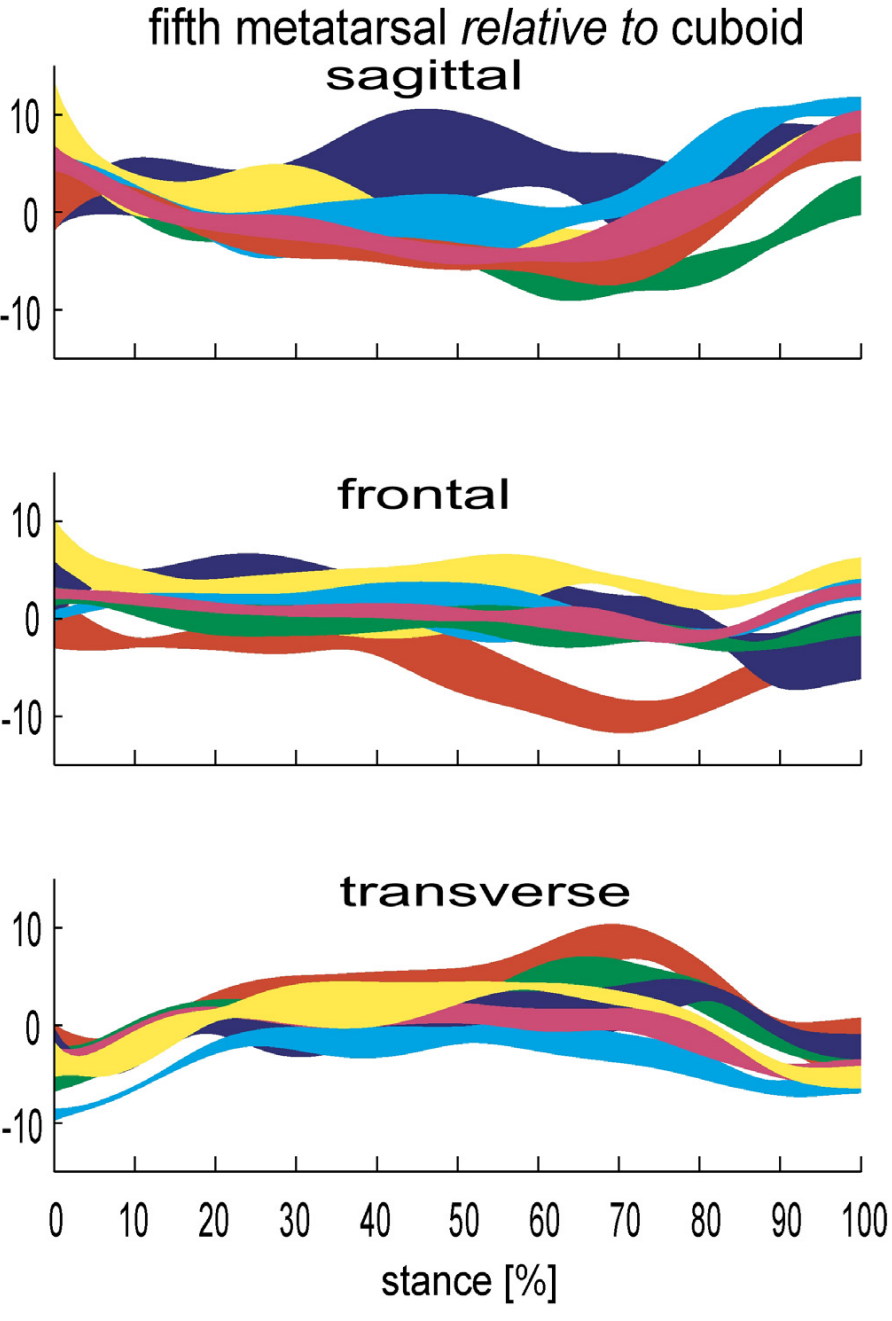
**5^th ^Metatarsal-cuboid kinematics for 6 subjects during stance (0–100%)**. Each band describes the mean +/- 1SD for each subject. +ve angles are plantarflexion, eversion and adduction of the talus relative to tibia. Motion in degrees(°).

For the forefoot, data have confirmed the greater stability of the first, second and third metatarsals compared to metatarsals four and five. The fourth and fifth metatarsals are functionally distinct from the other three metatarsals, in that they consistently displayed more motion during stance. Using a dynamic cadaver model, Nester et al [9] reported > 12° mean total range of motion in the sagittal and frontal planes between the fifth metatarsal and cuboid. These figures were broadly confirmed in subsequent invasive study (13.3° and 10.4° respectively [26]) (figure 5). Equivalent data for the other metatarsals was 5 to 8°.

Furthermore, the average total range of motion between the first metatarsal and medial cuneiform reported by Lundgren et al [26] was far less than the motion between the equivalent fifth metatarsal and cuboid (5.3°, 5.4° and 6.1° in the sagittal, frontal, transverse planes compared to 13.3°, 10.4° and 9.8°). This mobility on the lateral side of the foot is in addition to the motion between the cuboid and calcaneus (9.7°, 11.3° and 8.1° respectively) clearly demonstrating an infrequently discussed 'lowering' of the lateral arch of the foot.

There is an important observation from the slow running data reported by Arndt et al [27] and walking data from Lundgren et al [26], which is even more valuable since the data for the former study was collected on the same subjects and in the same session (day) as the latter study. The total range of motion at the subtalar, talonavicular, calcaneo-cuboid, cuboid-navicular, medal cuneiform-navicular, metatarsal 1-cunieform, and metatarsal 5-cuboid, was smaller in (slow) running than in walking. For the ankle, the range of motion during walking was far greater in the sagittal plane, and slightly greater in the frontal and transverse planes. Less foot motion suggests a stiffer structure, and given external forces are known to be greater during running, this suggests that greater muscle forces would be generated to control foot movements. One extrapolation from this observation is that foot orthoses for running need not be stiffer or have greater 'control' features (such as high levels of medial heel wedging) compared to orthoses for walking, since the motion taking place is already less.

## Conclusion

### Do we know how the foot really moves during gait?

Recent dynamic cadaver and invasive kinematic research has provided some useful insights. The rearfoot plays only a part of overall foot kinematics and we have consistently undervalued the contribution from mid- and forefoot articulations. This suggests that in order to control foot pronation, orthoses need to provide support across the entire rear- and mid foot and that the use of heel wedges alone is unlikely to produce the desired biomechanical effects on the foot. The forefoot undergoes a complex series of rotations which must influence the action of the intrinsic muscles of the foot, and researchers are only recently being able to investigate some of their functions [6].

Finally, variation between people in foot kinematics is high and normal. This includes variation in how specific joints move and how combinations of joints move. The foot continues to demonstrate its flexibility in enabling us to get from A to B via a large number of different kinematic solutions. Rather than continue to apply a poorly founded model of foot type whose basis is to make all feet meet criteria for the mechanical 'ideal' or 'normal' foot, we should embrace variation between feet and identify it as an opportunity to develop patient-specific clinical models of foot function. Clinicians should consider foot function in terms of the entire foot, and, given what we know about the variation between subjects, the general ranges of motion likely at specific joints, and what is observable clinically, rationalise the most likely kinematic solution for each patient. It is hoped that patient-specific conceptual models for foot biomechanics will lead to improved understanding of the role (if any) of foot biomechanics in causation of foot and lower limb problems, and improve our design of orthoses such that they have more precise and predictable biomechanical effects. With evidence of wide variation in foot kinematics from even small samples of participants, and of patient-specific response to orthoses [19], how can clinical practice continue to be so heavily based on the idea that one foot model should fit all, and that orthosis design and prescription is based on the ideal foot, rather than the dynamics of the foot of each patient?

## Competing interests

The author declares that they have no competing interests.

## Authors' contributions

The author is the sole writer of this paper. Contributions from prior research collaborations are identified under acknowledgements.

## Supplementary Material

Additional file 1 **Video 1 cadaver video**. The video illustrates the performance of the dynamic foot model. Click here for file

Additional file 2 **Video 2 bonepinvideo**. The video illustrates walking with the bone pins insitu. Click here for file
